# Centrifugal pipette-tip extraction using pyrolyzed cotton fibers combined with mass spectrometry: High-throughput analysis of methadone and its major metabolite in human urine

**DOI:** 10.1007/s00604-026-07937-4

**Published:** 2026-02-26

**Authors:** Jaime Millán-Santiago, Rafael Lucena, Soledad Cárdenas

**Affiliations:** Affordable and Sustainable Sample Preparation (AS2P), Departamento de Química Analítica, Instituto Químico para la Energía y el Medioambiente IQUEMA, Universidad de Córdoba, Campus de Rabanales, Edificio Marie Curie, Córdoba, E-14071 Spain

**Keywords:** Bioanalysis, Centrifugal-pipette tip micro-solid phase extraction, Sample preparation, Substrate spray mass spectrometry

## Abstract

A microextraction procedure is presented based on centrifugal-pipette tip microsolid phase extraction using pyrolyzed cotton fibers as sorbent to determine methadone and its major metabolite in urine samples by substrate spray mass spectrometry. The variables affecting the extraction and ionization of the analytes were studied, including the amount of sorptive phase (4 mg), sample volume (1 mL), washing volume (0.5 mL), centrifugation speed (4000 rpm), and eluent (acetonitrile/isopropanol, 1/1). The proposed analytical method was validated yielding limits of detection lower than 1.5 µg/L and demonstrating good linearity (R²> 0.988) up to 150 µg/L. Intra- and inter-day precisions, expressed as relative standard deviation (RSD) and calculated at four concentration levels, were better than 22.7%. Intra- and inter-day accuracy, expressed as relative recovery and calculated at four concentration levels, spanned from 69.5 to 125.3%. The centrifugal-pipette tip extraction allows the simultaneous processing of up to 12 samples in 8 min. This rapid extraction, combined with the short analysis time (< 2 min), provides a high-throughput bioanalytical method. Moreover, the novel microextraction device allows for the processing of up to 2 mL of sample, while the new ambient ionization mass spectrometry (AIMS) interface reduces the void volume, thereby speeding up the analysis time.

**Graphical Abstract:**

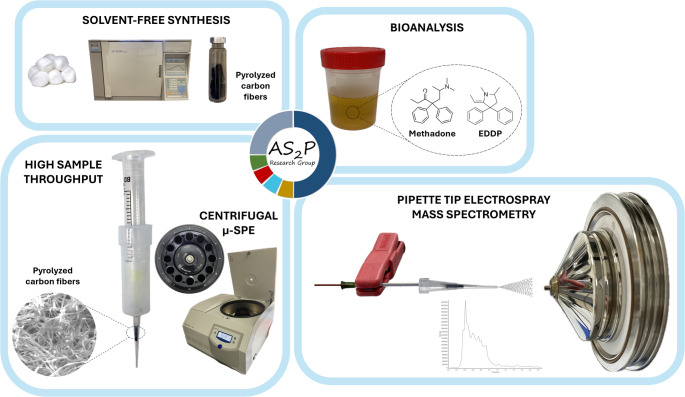

**Supplementary Information:**

The online version contains supplementary material available at 10.1007/s00604-026-07937-4.

## Introduction

Sample preparation is an essential step in many analytical procedures involving the analysis of complex samples [1]. In this sense, miniaturized extraction plays a crucial role in achieving the required sensitivity and selectivity levels while minimizing the environmental impact of conventional techniques. Modern sample preparation tends to simplify the analytical procedure in several ways: by (ultra)miniaturizing the techniques, designing high-throughput procedures [2, 3], eliminating the instrumental separation step [4], or integrating different steps of the analytical procedure. The latter trend has led to various strategies, including on-site extraction [5] and analysis [6], as well as on-line mass spectrometry [7].

Although the direct analysis of samples at ambient conditions speeds up the analysis by eliminating both sample preparation and chromatographic separation, it presents several drawbacks in terms of sensitivity, selectivity, and instrumental contamination attributed to the complexity of the sample matrices [8]. In this sense, coupling microextraction to ambient ionization mass spectrometry (µe-AIMS) [9] stands out as a versatile alternative, enabling the preconcentration of analytes and sample clean-up to reduce ion suppression and background noise, while maintaining a fast analysis time. Substrate spray mass spectrometry (SSMS) is a modality of AIMS that utilizes a sharp tip element, known as an electrospray emitter, as the analysis substrate. This element is positioned in front of the MS inlet and connected to a high-voltage electrical source. When a solvent is added to the substrate, it electromigrates to the tip, eluting the analytes. Due to the charge accumulation, an electrospray is formed at the tip of the emitter. µe-AIMS is a field that has been growing substantially during the last years as several miniaturized sorbent-based extraction techniques have been used, including solid phase microextraction [10], thin film microextraction [11], slug flow microextraction [12], µ-SPE [13], magnetic dispersive µ-SPE [14], or miniaturized stir bar sorptive dispersive microextraction [15]. In this context, a wide range of elements can be used as both microextraction devices and electrospray emitters, including coated blades [16], probes [17], needles [13], wooden tips [18], paper [19], aluminum foil [20, 21], or pipette tips (PTs) [22].

PTs are widely used in laboratories to handle solutions. The low cost of the devices makes them disposable, which is very convenient in bioanalysis as it reduces cross-contamination. The tips can hold a sorptive phase, giving rise to the so-called pipette tip extraction (PTE). Depending on how the sorptive phase is placed, two different workflows can be developed: (i) *dispersive pipette extraction* (DPX) in which a powder sorbent is introduced into the tip containing two distant frits, and it is dispersed when aspirating/dispensing the sample [23, 24], or (ii) *pipette tip micro-solid phase extraction* (PT-µ-SPE), in which the sorbent is packed inside the tip [25]. In both workflows, the tips are operated by micropipettes. Recently, our group has explored the potential of centrifugal PTE as a way of increasing the sample throughput using a conventional centrifuge, a common facility in most laboratories [22].

The applicability of PTE has been widely demonstrated for the analysis of complex matrices, such as biofluids [26], food samples [27], or environmental water samples [28]. The configuration of a PTE workflow is suitable for its direct hyphenation with analytical instrumentation [2], including mass spectrometry. Consequently, the development of this field is considerable, conferring high versatility to the technique, such as on-site extraction [22, 29], the use of portable instrumentation, including miniaturized mass spectrometry for on-site analysis [30], high throughput extraction using multichannel pipettes [31], or automated PTE [32].

In this article, a sustainable and rapid centrifugal-PT-µ-SPE is combined with AIMS for determining methadone and its major metabolite in urine samples. Pyrolyzed cotton fibers (py-CFs) are evaluated as the sorptive phase for isolating the compounds, considering their extraction capacity and their environmentally friendly origin. Pyrolyzed natural materials, in the form of biochar [33, 34] or carbon fibers [35], have been extensively used in the last decade in sample preparation. They are obtained from natural materials (cellulose or lignocellulose substrates) by an easy and almost solventless synthesis, although some modifiers can be added to adjust their chemical properties [36]. Therefore, they can be considered sustainable and affordable sorbents. As demonstrated, the polarity of these materials can be tuned by simply controlling the pyrolysis temperature and time, allowing the synthesis of phases with balanced polarity interactions [37]. In addition, a high sample throughput extraction based on PT-µ-SPE is presented by using a benchtop centrifuge, while the integration of the sample preparation step with the analysis of the samples by SSMS simplifies the analytical method.

## Materials and methods

### Reagents and samples

All the reagents used in this work were of analytical grade or better. Unless otherwise indicated, they were purchased from Sigma Aldrich (Madrid, Spain). Stock standard solutions of 2-ethylidene-1,5-dimethyl-3,3-diphenylpyrrolidine (EDDP) and methadone (MTD) were individually prepared at 1000 mg/L in methanol (Panreac, Barcelona, Spain) and stored at -22 °C. An intermediate working standard solution containing both analytes was prepared by diluting the stock standard solution to 10 mg/L in methanol and stored at -22 °C. Deuterated EDDP (EDDP-d_3_) and methadone (MTD-d_3_) were used as internal standards (ISTD). ISTD stock solutions were prepared at 10 mg/L in methanol and stored at -22 °C. Working solutions were prepared by spiking blank urine samples with the analytes as required. Cotton was purchased in a local supermarket (Córdoba, Spain). Ammonium hydroxide (30%, v/v) (Panreac, Barcelona, Spain) was used to adjust the pH of the samples. Formic acid (≥ 98%), Milli-Q water (Millipore Corp., Darmstadt, Germany), and methanol were used as carrier phase for direct infusion tandem mass spectrometry (DI-MS/MS) analysis, while acetonitrile (Panreac, Barcelona, Spain) and 2-propanol were used as eluent in pipette tip electrospray ionization tandem mass spectrometry (PT-ESI-MS/MS).

Blank urine samples were obtained from four healthy volunteers (two males and two females) who were not consuming any of the analytes studied in this article, and collected in sterile sample containers (Deltalab, Barcelona, Spain). A pool of samples, provided by both male and female volunteers, was used for the study of the variables and method validation. The samples were adjusted at pH 10 with ammonium hydroxide and subsequently centrifuged at 11,068 *g* for 5 min prior to the extraction procedure. Real urine samples, obtained from volunteers (Samples 1–4: two males and two females) from the Addiction Unit (Instituto Provincial de Bienestar Social, Córdoba, Spain) and a patient undergoing a surgery (Sample 5: female) were stored at -80 °C until analysis.

### Synthesis and characterization of py-CFs

Py-CFs were prepared under a gradient temperature in an oven, without the necessity of any other reagent involved in the synthesis. 350 mg of cotton was introduced in a 20 mL headspace vial sealed with a gas chromatography septum cap. The cotton fibers were heated following a four-segment isocratic program. During each segment, the fibers were heated at a constant temperature (200 °C, 250 °C, 300 °C, 325 °C) for one hour. The septum was pierced approximately every 30 min with a hypodermic needle to release the gas and prevent system overpressure. The obtained py-CFs were transferred to a 50 mL Falcon tube and washed sequentially with 25 mL of methanol and water for 15 min at 1000 rpm using an orbital stirrer. Finally, the py-CFs were dried overnight at room temperature and stored until their use.

Scanning electron microscopy (SEM) images were obtained using a JEOL JSM 7800 microscope. Raw cotton and py-CFs were coated with gold to improve the conductivity and the quality of the SEM images. Carbon and hydrogen content of raw cotton and py-CFs was determined in a EuroVector Elemental Analyzer EA3000 (EuroVector SpA, Milan, Italy). Both characterization techniques were carried out in the Central Service for Research Support (SCAI) of the University of Córdoba.

### Centrifugal-PTE procedure

**Fig. 1:**
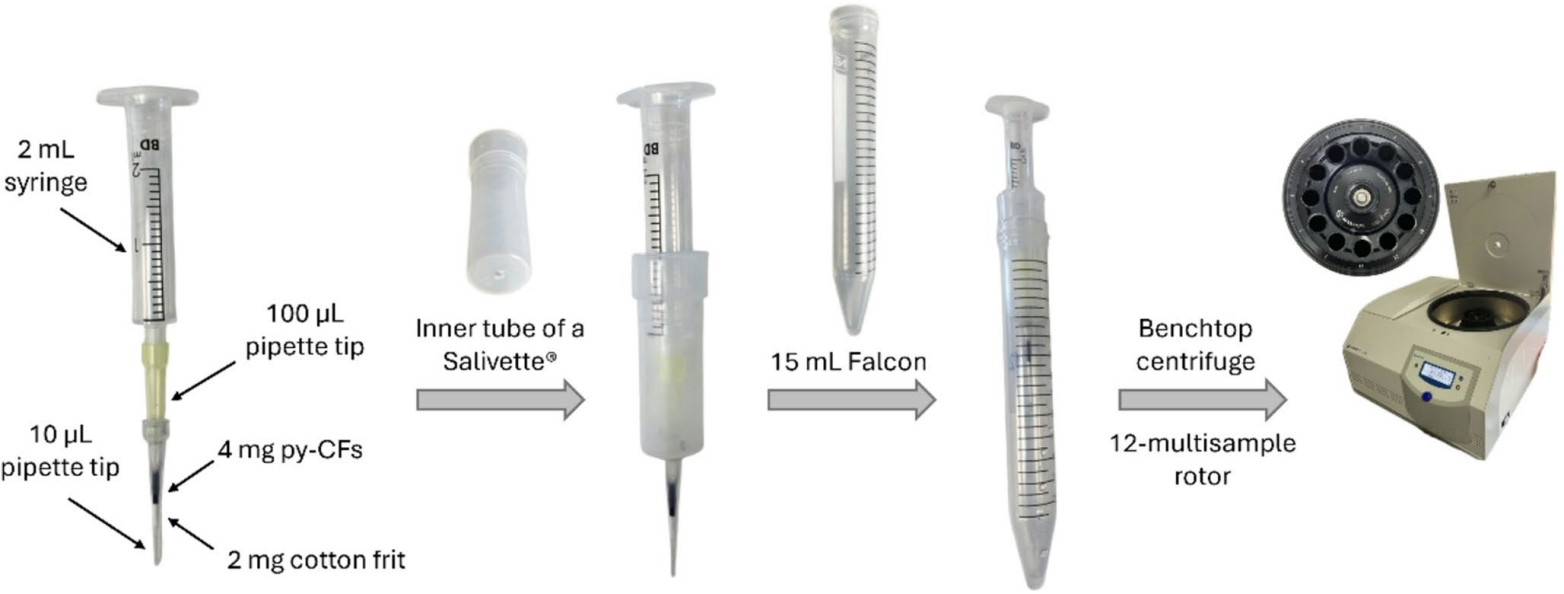
Scheme of the extraction unit, where the different elements and how they assemble are shown. For details, see the text

The extraction device, as shown in Fig. 1, consists of a 10 µL PT where 4 mg of py-CFs are packed in the base of the tip using a cotton frit (2 mg) to prevent any loss of the sorptive phase during extraction. The PT containing the sorbent is attached to a 2 mL syringe sample holder using a precut 100 µL PT as an adapter. The resulting unit is attached to the inner tube of a Salivette sampler (Sarstedt, Nümbrecht, Germany) and incorporated into a 15 mL Falcon tube used as a solvent reservoir. This final device is compatible with a conventional centrifuge, which is used to force the flow of the sample and the different solvents through the sorptive phase. A Sigma 3-16KL refrigerated benchtop centrifuge (Sigma Laborzentrifugen GmbH, Osterode am Harz, Germany), equipped with a 12311 rotor, is used allowing the simultaneous extraction of 12 samples. Each step of the extraction procedure involves centrifuging the extraction device at 1774 *g* for 2 min. Firstly, the py-CFs are sequentially conditioned and equilibrated with 500 µL of methanol and Milli-Q water (pH 10), respectively. In the next step, 1 mL of undiluted urine, adjusted to pH 10, is passed through the sorptive phase to retain the target analytes. Finally, 500 µL of Milli-Q water (pH 10) is added to remove potential co-extracted interferences.

### PT-ESI-MS/MS analysis

PT-ESI-MS/MS analysis was performed according to the previous experience of the group in substrate spray mass spectrometry using needles and PTs [9]. The proposed interface is presented in Figure S1. The 10 µL PT containing the py-CFs, which acts as ESI emitter, is placed in front of the MS inlet, leaving a distance of 7 mm. A 16-gauge blunt needle is used as an electrode (connected to an electric clamp) to provide a 5 kV electrical gradient, which is essential for generating the electrospray. At the same time, the hollow needle permits the flow of the eluent (acetonitrile/isopropanol, 1:1, v/v) provided by a syringe pump at a constant rate of 30 µL/min through a PEEK tube. During the 1 min length elution, the MS/MS signal profile of the samples is acquired under the conditions described in the supplementary information (Table S1).

## Results and discussion

In a previous study, our research group proposed the so-called centrifugal-PT-µSPE as a rapid alternative for processing multiple samples simultaneously [22]. This article aimed to deepen the potential of the technique and simplify the overall analytical procedure by exploiting our experience in hyphenating extraction techniques to AIMS. The extraction device proposed in this article has been adapted to increase the sample load five times, opening the door to process urine samples where the analytes are typically diluted compared to saliva. Also, a fibrous sorptive phase is employed to facilitate the rapid and efficient isolation of the target analytes. Carbon fibers, obtained by the pyrolysis of cotton, have been selected due to their balanced polarity (which depends on the synthetic conditions) that is essential to retain in a single step a non-polar drug and its polar metabolite. Instead of the classical offline extraction-elution-analysis workflow, the tips with retained analytes have been directly analyzed by AIMS using a dedicated interface that combines conductor needles and plastic PTs [38]. For this purpose, a 10 µL PT has been used to hold the sorbent. The narrow size of the tip has been demonstrated to be essential for creating a stable spray during AIMS analysis, while the location of the sorbent focuses the sample plug and increases sensitivity.

### Characterization of the sorptive phase

**Fig. 2:**
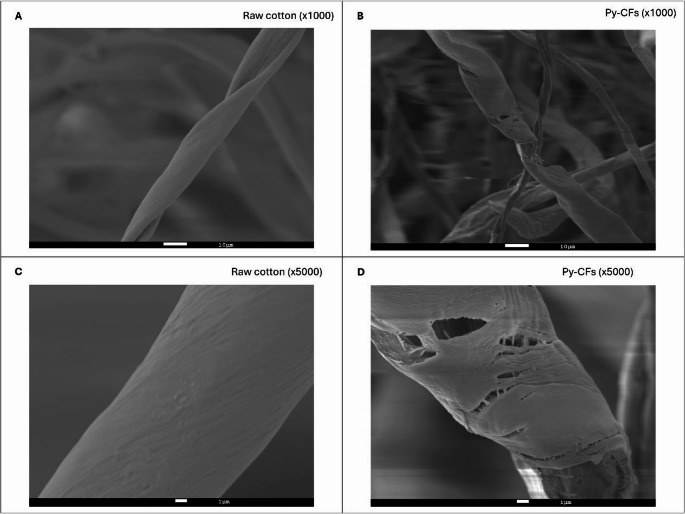
SEM images of (**A**) raw cotton at 1000 magnifications, (**B**) py-CFs at 1000 magnifications, (**C**) raw cotton at 5000 magnifications, and (**D**) py-CFs at 5000 magnifications

Raw cotton and py-CFs were characterized by SEM to evaluate the geometry of the fibers before and after the pyrolysis. Raw cotton presents a smooth fiber-shaped geometry typically associated with its cellulosic composition (Fig. 2A and C), while py-CFs obtained following the temperature program up to 325 °C present a fibrous structure with pores and irregularities (Fig. 2B and D). This change can be attributed to the decomposition of the fibers due to carbonization, which is also evident at a macroscopic level, as the py-CFs are more fragile compared to raw cotton.

Carbon and hydrogen content of raw cotton and py-CFs were measured to evaluate the differences associated with the pyrolysis step. The amount of C increased from 42.8% of raw cotton to 71.8% of py-CFs, while the amount of H decreased from 7.5 to 4.9%. The increase in carbon content is typically associated with an increase in hydrophobic character. For this reason, the conditions selected for this synthesis result in a sorptive phase with a balanced polarity [39], which is essential considering the logD (3.9 for MTD and 3.8 for EDDP) of the analytes. Consequently, the expected interactions between the py-CFs and the analytes are mostly hydrophobic, including van der Waals forces and π-π interactions, although hydrogen bonds can also be established to a lesser extent.

The spectra for raw cotton and py-CFs are represented in Figure S2. As can be observed, the characteristic bands of the cellulose are almost lost due to the thermal treatment. Two slight signals at ca. 1704 and 1590 cm^− 1^ are observable. These bands can be ascribed to a carbonyl group and a C = C (stretching) in a cyclic structure. The carbonyl groups may develop H-bonding with the analytes.

#### Study of the variables affecting the extraction efficiency of the analytes

The variables affecting the extraction efficiency of the analytes were investigated using blank urine fortified with the analytes at a concentration of 50 µg/L. Each condition was evaluated in triplicate. In these studies, direct infusion tandem mass spectrometry (DI-MS/MS) was employed as the instrumental technique (Table S2).

**Fig. 3:**
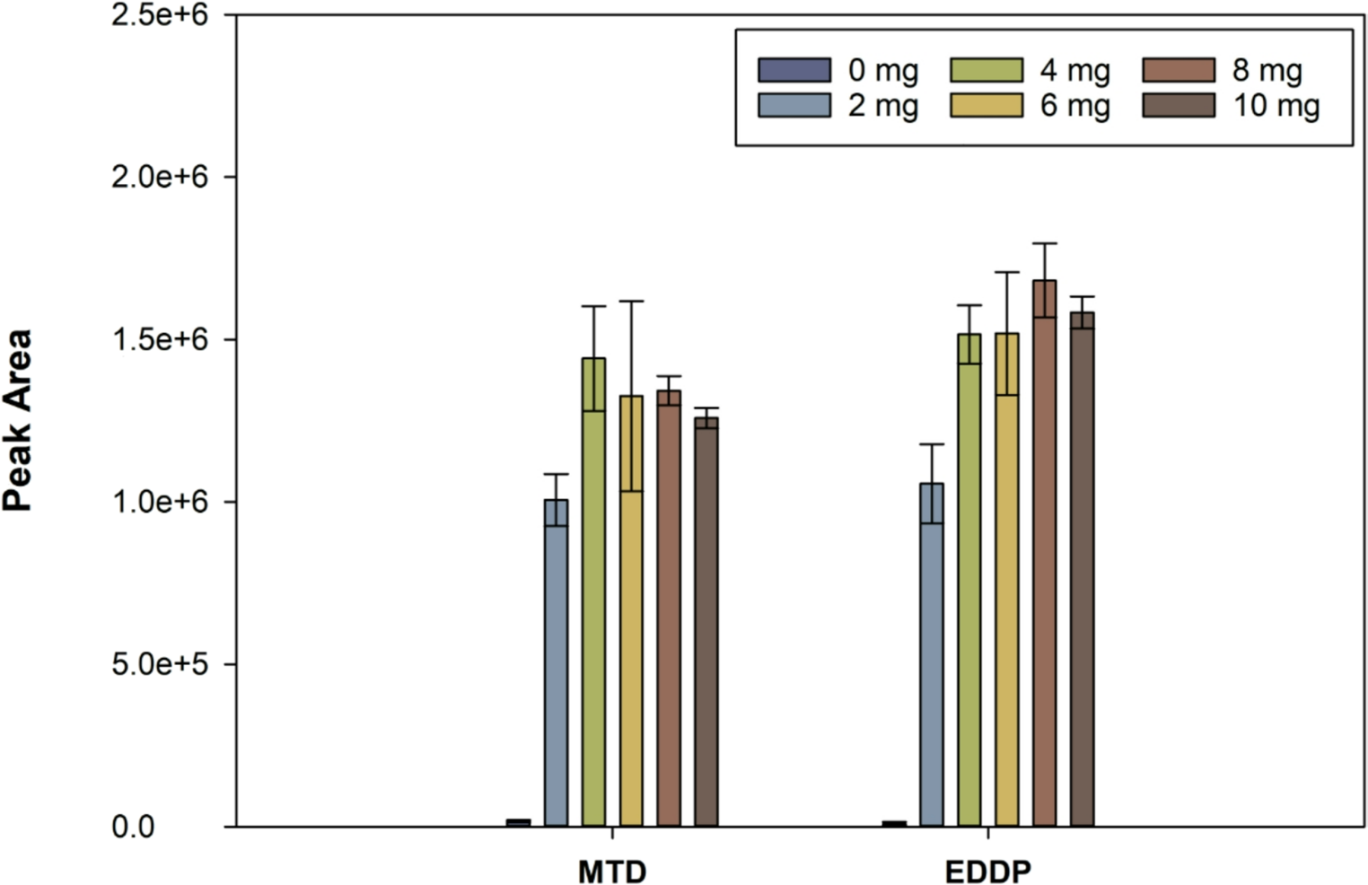
Effect of the amount of py-CFs on the extraction of the analytes

Thepy-CFs amount was studied in the interval from 0 to 10 mg, using a 2 mg frit of raw cotton to avoid losses of the sorbent material. For this purpose, several tips containing different amounts of py-CFs (0, 2, 4, 6, 8, and 10 mg) were fabricated. The results, presented in Fig. 3, demonstrate the active role of py-CFs in the extraction of analytes as the presence of py-CFs increases the extraction efficiency 87 and 165 times for MTD and EDDP, respectively, compared to raw cotton. The steady state in the extraction step is achieved at 4 mg of sorptive phase, where an asymptote is almost reached, considering the standard deviations. For this purpose, 4 mg of py-CFs were selected as optimum to promote the flow of the sample and solvents and minimize any overpressure risk.

**Fig. 4:**
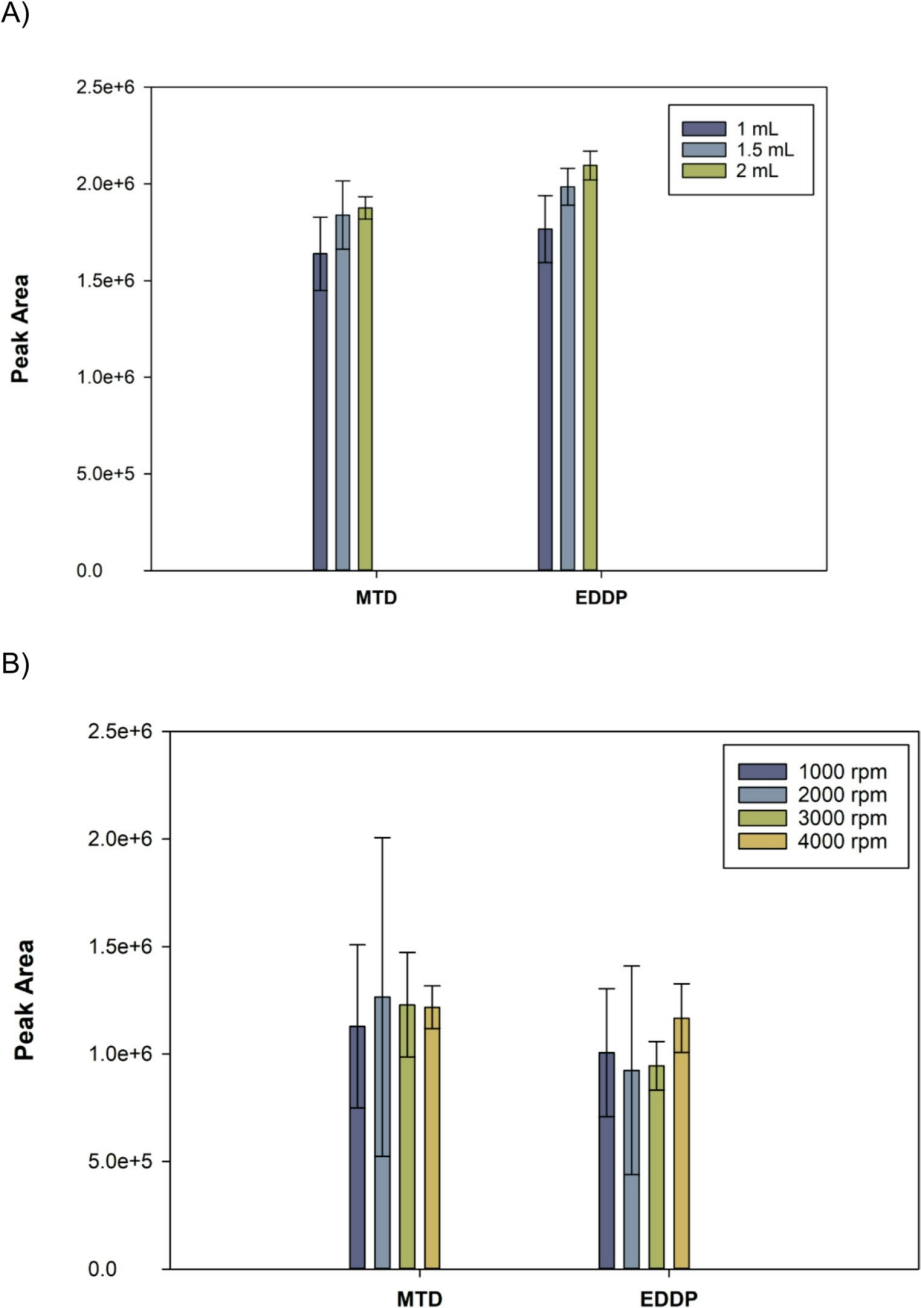
Effect of (**A**) the sample volume and (**B**) centrifugation speed on the analytical signal for the analytes

The sample volume was studied between 1 and 2 mL. Larger volumes were not considered, as 5 mL syringes did not fit into the 15 mL Falcon tube. Figure 4A shows a slight enhancement of the analytical signal with the sample volume, although this enhancement was not proportional to the increasement of the sample volume. Consequently, the sample volume was set at 1 mL to facilitate the handling of samples and the extraction units. To remove potential coextracted compounds, the clean-up step was also studied varying the volume of the washing solution (Figure S3). According to the results, 0.5 mL was selected as the optimum value.

The centrifugation speed was optimized to ensure the flow of the entire sample through the sorbent in the shortest time, while maintaining adequate reproducibility and sensitivity. The studied speed values were 1000 rpm (111 *g*), 2000 rpm (443 *g*), 3000 rpm (996 *g*) and 4000 rpm (1771 *g*). The centrifugation speed is inversely proportional to the time necessary to guarantee the complete flow of the sample (Figure S4). For example, at the lowest speed (1000 rpm), it took 17 min to process the entire sample, whereas at 3000 and 4000 rpm, only 2 min were required. Regarding the extraction efficiency of the analytes (Fig. 4B), it remains constant at different centrifugation rates. However, the reproducibility is positively affected at higher rates, which can be attributed to a more efficient flow of the sample. Consequently, 4000 rpm was selected as the centrifugation speed.

#### 3.2.2 Study of the variables affecting the direct combination PT-ESI-MS/MS

**Fig. 5:**
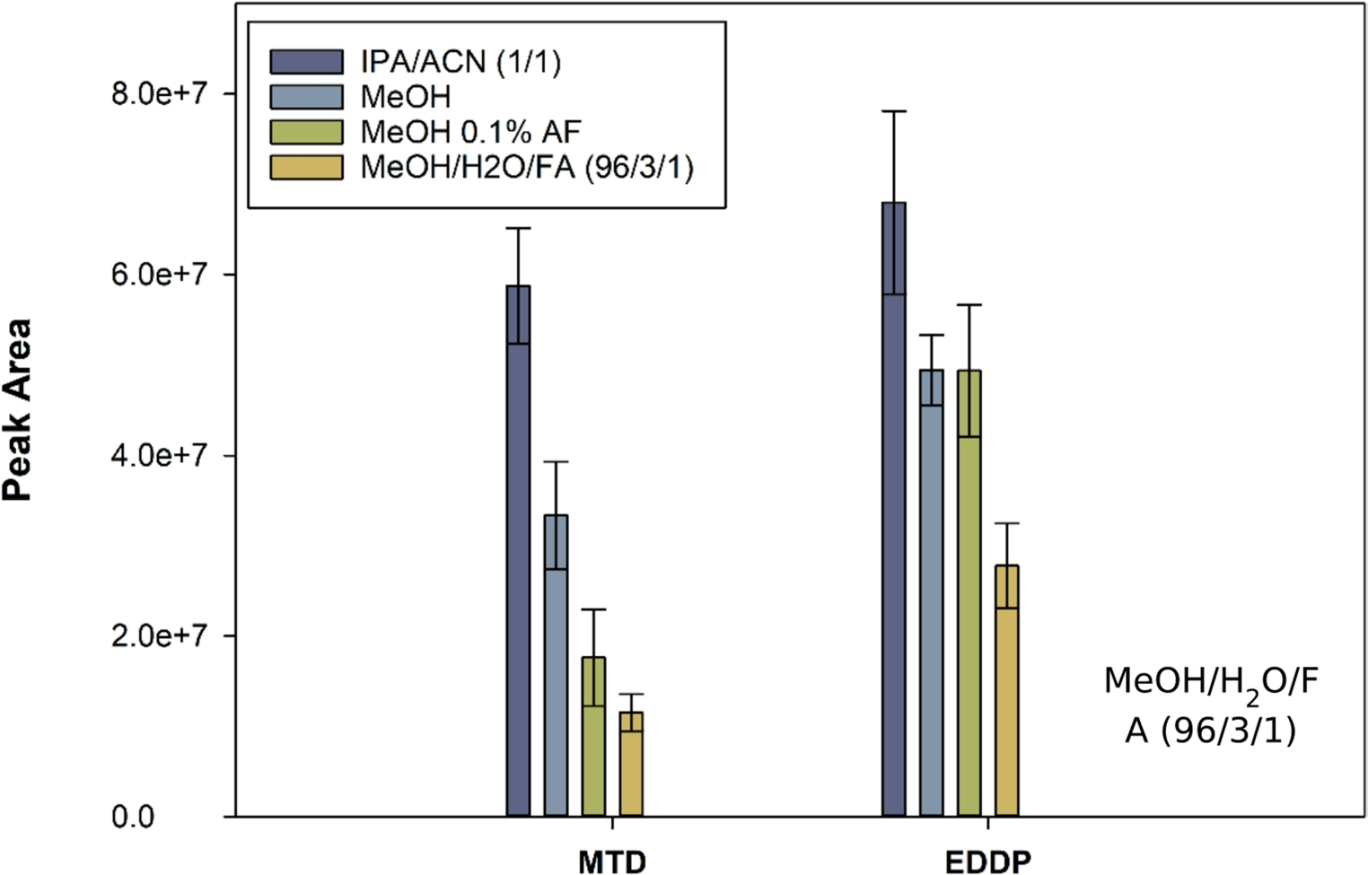
Effect of the eluent on the analytical signal obtained by PT-ESI-MS/MS

PT-ESI-MS/MS is affected by different variables, including the applied voltage, the distance between the tip and the MS inlet, the eluent flow rate, and the type of eluent. The first three parameters were fixed according to our previous experience since they are not affected by the type of sorptive phase used. The type of eluent, however, is critically dependent on the material used for isolating the analytes, as the eluent must efficiently break the sorbent-analyte interaction. Additionally, it serves as the ESI medium, which must favor the ionization of the analytes. Four different eluents have been studied, including isopropanol/acetonitrile (IPA/CAN, 1:1, v/v), methanol, methanol containing 0.1% (v/v) formic acid, and methanol/water/formic acid (96:3:1, v/v). The results are shown in Fig. 5. IPA/ACN provides the best sensitivity for both MTD and EDDP, while the methanol/water/formic acid mixture presents the worst results. This behavior can be associated with the presence of water, which may negatively affect the evaporation of the solvent. As an example, the elution profiles obtained for EDDP are shown in Figure S5.

### Method validation

The proposed analytical method was validated following the ICH guideline M10 on bioanalytical method validation [40]. The figures of merit are presented in Table 1. The limits of detection (LODs), limits of quantification (LOQs), linear range, and linearity were calculated using a matrix-matched calibration curve prepared from a pool of blank urine samples from both male and female donors to take into account the variability associated to the different individuals. The LODs and LOQs were calculated as 3 and 10 times the signal-to-noise ratio, respectively. The LODs were 1.5 for MTD and EDDP and the linear range spanned from 5 to 150 for MTD and from 5 to 200 for EDDP, while the linearity was R^2^ > 0.988 in both cases.

**Table 1:** Analytical figures of merit obtained following the ICH guideline M10 on bioanalytical method validation

Analyte	LOD (µg L^− 1^)	LOQ (µg L^− 1^)	Linear range (µg L^− 1^)	Linearity (*R*^2^)	Intra-day precision ^a^ (RSD, %)				Intra-day accuracy ^a^ (RR, %)				Inter-day precision ^b^ (RSD, %)				Inter-day accuracy ^b^ (RR, %)				Dilution integrity ^a^ (10 µg L^− 1^)	
					LLOQ	LQC	MQC	HQC	LLOQ	LQC	MQC	HQC	LLOQ	LQC	MQC	HQC	LLOQ	LQC	MQC	HQC	Precision (RSD, %)	Accuracy (RR, %)
MTD	1.5	5	5-150	0.988	14.1	13.1	22.7	17.9	122.8	97.9	69.5	71.4	5.9	4.8	6.7	13.5	121.2	90.9	73.2	96.9	21.0	90.2
EDDP	1.5	5	5-200	0.993	13.9	8.1	8.7	4.5	125.3	118.0	100.2	92.2	12.7	11.7	1.0	7.8	115.8	100.3	93.6	92.5	13.1	119.9

*LOD* limit of detection, *LOQ* limit of quantification, *LLOQ* lowest limit of quantification, *LQC* low quality control, *MQC* medium quality control, *HQC* high quality control, *RDS* relative standard deviation, *RR* relative recovery

^a^: *n* = 5, ^b^: *n* = 3

Inter-day (*n = 3*) and intra-day (*n = 5*) accuracy and precision, expressed as relative recovery (RR, %) and relative standard deviation (RSD, %), respectively, were calculated using different independent pools of blank urine samples spiked at four concentration levels: lowest limit of quantification (LLOQ), low quality control (LQC), medium quality control (MQC), and high quality control (HQC). The LLOQ, LQC, MQC and HQC concentrations are 5, 10, 50, and 100 µg/L for MTD, while for EDDP were 5, 10, 100, and 200 µg/L. Intra-day and inter-day precision was better than 22.7% and 13.5%, respectively, for MTD, while for EDDP was better than 13.9% and 12.7%, respectively. Intra-day and inter-day accuracy spanned from 69.5% to 122.8% and from 73.2% to 121.2%, respectively, for MTD, while for EDDP ranged from 92.2 to 125.3% and 92.5-115.8%, respectively.

The dilution integrity of the proposed method was evaluated by spiking a blank urine sample (*n = 5*) at a concentration five times higher than the upper limit of quantification (ULOQ) and diluting it to a concentration of 10 µg/L while maintaining the concentration of ISTD. The precision was better than 21.0%, while the accuracy ranged from 90.2% to 119.9% for both analytes.

The stability of the analytes in the sorptive phase was studied (Figure S6). Twelve different replicates from the same sample, previously spiked at 50 µg/L, were extracted and stored at room temperature in the dark. Three different sorptive phases were eluted and analyzed by PT-ESI-MS/MS after 4, 7, 11, and 14 days. The reproducibility between days yielded RSD values better than 18.9% and 4.4% for MTD and EDDP, respectively. This demonstrates the potential of the proposed method to carry out the extraction step in a conventional laboratory and then sending the sorptive phases to central or reference laboratories to carry out the PT-ESI-MS/MS analysis.

### 3.4 Analysis of samples from patients under methadone treatment

Five different samples obtained from patients under methadone treatment were analyzed under the proposed analytical method. Each sample was analyzed in triplicate. Samples 1–3 presented a concentration lower than the LOD of both analytes. Samples 4 and 5 presented a concentration of 142 ± 33 and 74 ± 10 µg/L for MTD, while EDDP concentration was above the ULOQ in both cases. A chronogram obtained from the analysis of a single real sample is presented in Figure S7.

### Comparison with other methods

The comparison of the proposed method with other methods reporting the determination of methadone and EDDP in urine samples is presented in Table 2. In terms of sensitivity, the proposed analytical method provides LOQs that are higher than [41–46], while [47] presents LOQs higher than the proposed method. Following a validation guideline such as the ICH guideline M10 on bioanalytical method validation ensures the quality of the results at the expense of obtaining higher LODs and LOQs. The simultaneous extraction of the samples proposed by [41, 43, 44] and this method speeds up the sample throughput analysis. However, only [41, 45] presents a faster time to carry out the extraction procedure (< 5 and 2 min, compared to 8 min). Those methods that do not involve any instrumental separation step prior to sample analysis, such as direct infusion [41] or SPME-nano-ESI interface [45], are comparable to the proposed PT-ESI method, which achieves 2 min of analysis per sample. Consequently, solvent consumption and associated costs are reduced. However, analytical methodologies that require chromatographic separation [42–44, 46, 47] extend the analysis time from 8.5 to 34 min. In terms of greenness, the proposed method uses a sorptive phase obtained from a natural material (cotton) and the synthesis is solvent-free as it only requires the use of heat, while only [41] reports the use of recycled polymer.

**Table 2:** Comparison with other methods reporting the extraction of methadone or EDDP in urine samples

Sorbent	Extraction technique	Instrumental technique	Analytes	LOQ (µg/L)	Simultaneous extraction	Time of extraction procedure (min)	Time of analysis (min)	Reference
Recycled PS	In-syringe µSPE	DI-MS/MS	MTD	2.4	Yes; 25	< 5	2	[41]
SCX	SPE	LC-MS/MS	MTD	2.5	n.a.	n.a.	24	[42]
	DLLME			1				
C8-CN stationary phase	TFME	LC-MS/MS	MTD	3	Yes; 192	330	13	[43]
Porous sorptive polymer	TFME	UHPLC-MS/MS	MTD	0.05	Yes; 30	11	8.5	[44]
C18-SCX	SPME	Nano-ESI	MTD	0.1	n.a.	2	2.5	[45]
SCX	SPE	LC-MS/MS	MTD	0.05	n.a.	n.a.	34	[46]
			EDDP	0.05				
Mix-mode	SPE	HPLC-ITMS	MTD	100	n.a.	n.a.	22	[47]
			EDDP	100				
Py-CFs	Centrifugal-PT-µSPE	PT-ESI-MS/MS	MTD	5	Yes; 12	8	2	This work
			EDDP	5				

*PS* polystyrene, *μ-SPE* micro-solid phase extraction, *DI-MS/MS* direct infusion tandem mass spectrometry, *C8-CN* octyl-cyanopropyl, *LC-MS/MS* liquid chromatography tandem mass spectrometry, *SCX* strong cation exchange, *TFME* thin film microextraction, *UHPLC-MS/MS* ultra-high performance liquid chromatography tandem mass spectrometry, *SPME* solid phase microextraction, *ITMS* ion trap, *Py-CFs* pyrolyzed carbon fibers, *PT-μ-SPE* pipette tip-micro solid phase extraction, *n.a.* not available

### Evaluation of the greenness and practicality

The greenness and practicality of the proposed analytical method were evaluated using *Sample preparation metric of sustainability* (SPMS) [48], *Analytical greenness metric for sample preparation* (AGREEPrep) [49], and *Blue applicability grade index* (BAGI) [50], respectively. The pictograms with the overall results are shown in Figure S7. According to the SPMS tool (Figure S8A), the overall result (8.84 out of 10) demonstrates the grenness character of the analytical methodology, mostly based on the low sample, extractant, and waste amount, the natural origin of the extractant, and the possibility of extracting different samples simultaneously. However, the debilities lie in the number of steps and the time necessary to complete the extraction procedure, as well as the necessity to stir the samples by centrifugation. The AGREEPrep tool (Figure S8B) provides an overall result of 0.53 out of 1. The main strengths are the lack of hazardous materials involved in the analytical process, the reduced sample volume, the high sample throughput, and the operator safety. On the other hand, the main limitations are the *ex situ* sample preparation although an integrated elution-ionization is carried out and the advanced instrumentation that is required. In the case of BAGI (Figure S8C), the overall result (62.5 out of 100) is acceptable. In this case, the main advantages are the no need to preconcentrate the eluates and the high sample throughput. However, the limitations are related to the reduced number of analytes, the semi-automation of the process, and the necessity of instrumentation that is not available in all laboratories.

## Conclusions

In this article, an interface for substrate spray mass spectrometry is presented, utilizing PTs as microextraction devices and emitters to form an electrospray containing the analytes. This interface is employed to determine methadone and its major metabolite in human urine samples. The proposed interface is improved compared to previous reported by the research group in several ways: (i) the location of the sorbent inside the tip of the PT reduces the dead volume that leads to a shorter time of analysis, (ii) the selected eluent enhances the stability of the spray as the fluctuations are reduced, and (iii) the eluent provides a higher elution efficiency, allowing the analysis of the samples in less than 2 min.

The required elements to carry out the sample preparation (e.g., benchtop centrifuge, disposable syringes, and PTs) and the py-CFs sorbent, which uses cotton as a precursor, are affordable and easy to find in any laboratory. The synthesis of the py-CFs is a solvent-free process that only requires the use of a conventional oven, thereby conferring a sustainable component to the proposed analytical method. Moreover, the conditions can be easily controlled, ensuring reproducibility between batches. The low-cost of each extraction unit enables its disposability, thereby avoiding cross-contamination and reducing risk for the operator when handling biological samples.

In terms of the centrifugal-PT extraction device, the use of 2 mL disposable syringes to host the sample and solvents involved in the centrifugal steps increases the sample volume up to 5 times compared to the previous device. Although the extraction procedure is semi-automated, the main drawback of this methodology is the lack of automation in the analysis step, which necessitates the presence of an operator for each sample analysis. Moreover, the reduced number of analytes that are determined or the necessity of sophisticated instrumentation that is not available in all laboratories can also be identified as potential shortcomings.

## Supplementary Information

Below is the link to the electronic supplementary material.

Supplementary Material 1

## Data Availability

The authors declare that the data supporting the findings of this study are available within the paper and its SupplementaryInformation files. Should any raw data files be needed in another format they are available from the corresponding author upon reasonable request.
