# Supplementary material for: Centrifugal pipette-tip extraction using pyrolyzed cotton fibers combined with mass spectrometry: High-throughput analysis of methadone and its major metabolite in human urine
Source: Mikrochim Acta. 2026 Feb 26;193(3):195. doi: 10.1007/s00604-026-07937-4 (PMC12945943; doi:10.1007/s00604-026-07937-4)
Supplement: Supplementary file 1 — Supplementary Material 1 [file 604_2026_7937_MOESM1_ESM.docx]

**Centrifugal pipette-tip extraction using pyrolyzed carbon fibers combined with mass spectrometry: High-throughput analysis of methadone and its major metabolite in human urine**

Jaime Millán-Santiago,* Rafael Lucena, Soledad Cárdenas*

Affordable and Sustainable Sample Preparation (AS_2_P) research group, Departamento de Química Analítica, Instituto Químico para la Energía y el Medioambiente IQUEMA, Universidad de Córdoba, Campus de Rabanales, Edificio Marie Curie, E-14071, Córdoba, Spain.

Corresponding authors´ emails: [q52misaj@uco.es](mailto:q52misaj@uco.es) (J. Millán-Santiago); [qa1caarm@uco.es](mailto:qa1caarm@uco.es) (S. Cárdenas)

**1.1 PT-ESI-MS/MS**

*1.1 PT-ESI-MS/MS interface*

| A)  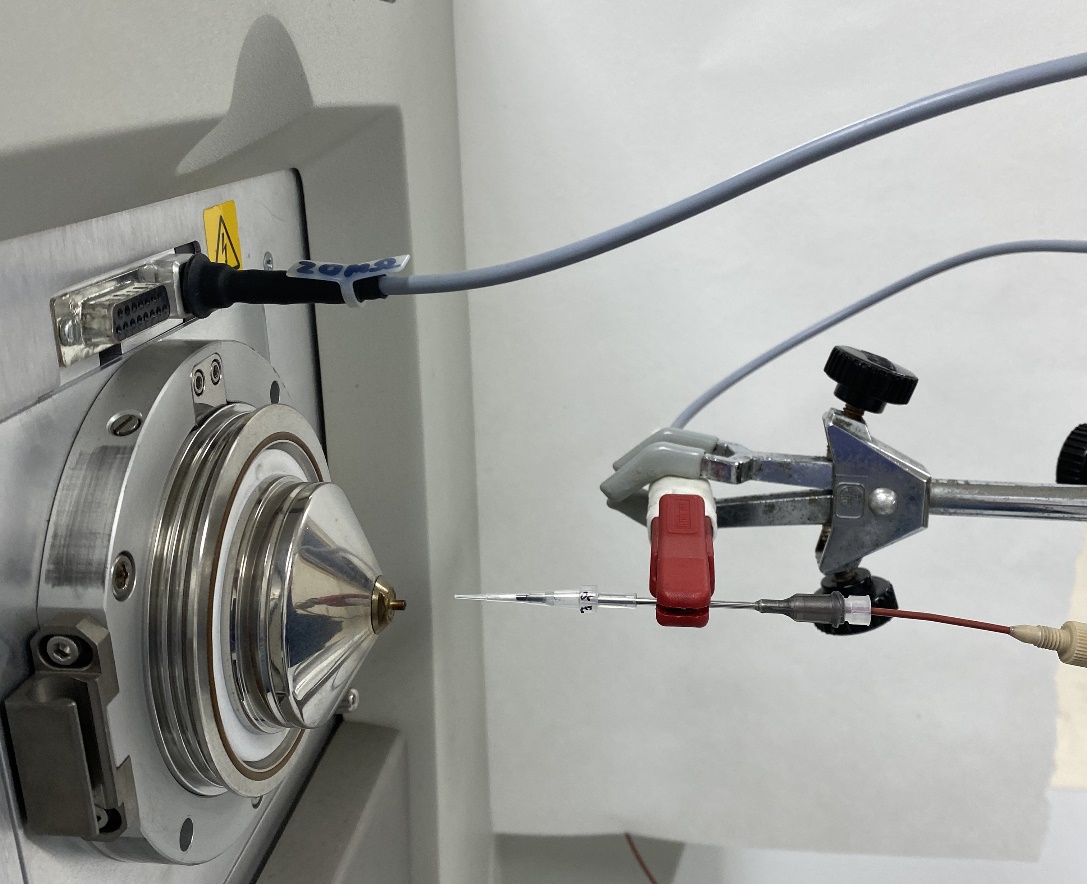 |
| --- |
| 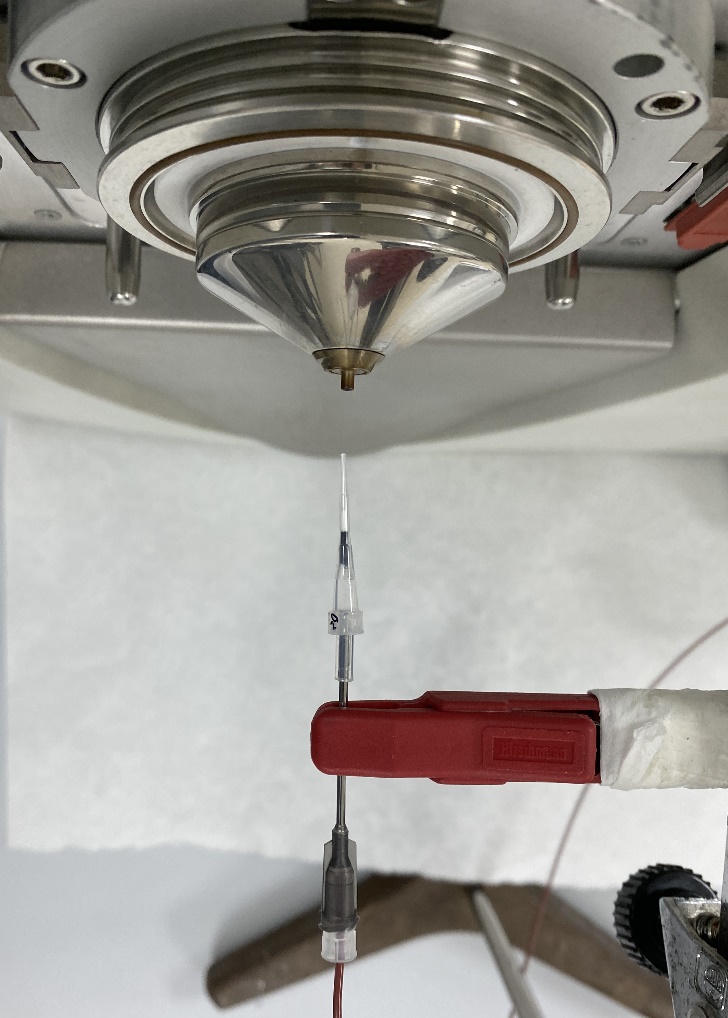B) |

**Figure S1**. PT-ESI-MS/MS proposed interface.

*1.2 PT-ESI-MS/MS analysis and multiple reaction monitoring parameters*

PT-ESI-MS/MS experiments were performed in a Thermo LTQ hybrid mass spectrometer (Thermo Fisher Scientific, San José, CA, USA) using the ion trap analyzer. The ionization parameters were as follows: the capillary voltage was 35 V, the capillary temperature was 275 °C, and the tube lens was 130 V. The data-dependent MS/MS events were performed on the most intense ions detected in positive mode full scan MS and the MS/MS isolation width was 1 amu. Tandem mass spectrometry (MS/MS) experiments were carried out by collision-induced dissociation (CID) using helium as the collision gas. Table S1 shows the MRM parameters for PT-ESI-MS/MS analysis. The activation Q was set at 0.250, while the activation time was set at 30 ms for all the analytes and ISTD. Data was collected and analysed with Xcalibur 2.1.0 software (Thermo Fisher Scientific).

**Table S1**. MRM parameters for pipette tip-electrospray tandem mass spectrometry.

| Analyte | Parent mass (m/z) | Product ion (m/z) | Normalized collision energy (%) |
| --- | --- | --- | --- |
| EDDP | 278.2 | 249.1 | 30 |
| EDDP-d_3_ | 281.2 | 249.1 | 35 |
| MTD | 310.3 | 265.2 | 30 |
| MTD-d_3_ | 313.3 | 268.2 | 30 |

**2. DI-MS/MS analysis**

The offline elution of the analytes that was carried out in the study of the variables consisted of attaching a 100 μL pipette tip to the 10 μL pipette tip containing the py-CFs. 100 μL of the eluent was added to the tip and a 2 mL disposable syringe was connected to the 100 μL pipette tip to force the flow of the eluent through the sorbent. The eluate containing the analytes was collected in an insert contained into a HPLC vial. 5 μL of the eluate was analyzed by DI-MS/MS on an Agilent 1260 Infinity HPLC system (Agilent, Palo Alto, CA, USA) equipped with a binary high-pressure pump for mobile phase delivery and an autosampler. A guard column (0.2-μm filter, 2.1 mm) was employed to prevent the MS from potential particles. The carrier phase consisted of A) Milli-Q water containing 0.1% formic acid and B) methanol containing 0.1% formic acid in a 10/90 proportion, and the flow rate was maintained at 0.2 mL min^-1^. Each analysis run takes 1.5 min to complete. Identification and quantification were performed on an Agilent 6420 Triple Quadrupole MS with an electrospray source. The flow rate and the temperature of the drying gas (N_2_, 99% purity) were 6 L min^-1^ and 350 ºC, respectively. The nebulizer pressure was 30 psi, and the capillary voltage was kept at 2500 V in positive mode. The multiple reaction monitoring (MRM) transitions for DI-MS/MS are described in Table S2. Agilent MassHunter Software (Version B.06.00) was used for qualitative and quantitative analyses.

**Table S2.** MRM parameters for DI-MS/MS analysis.

| Analyte | Precursor ion (m/z) | Product ion (m/z) | Fragmentor (V) | Collision energy (V) |
| --- | --- | --- | --- | --- |
| EDDP | 278.1 | 249.1 (Q) | 114 | 25 |
|  |  | 234.0 |  | 33 |
| EDDP-d_3_ | 281.2 | 249.1 (Q) | 120 | 26 |
|  |  | 234.0 | 120 | 30 |
| MTD | 310.2 | 105.1 | 114 | 34 |
|  |  | 265.1 (Q) |  | 14 |
| MTD-d_3_ | 313.2 | 105.1 | 114 | 34 |
|  |  | 268.1 (Q) |  | 14 |

(Q): quantification transition.

**3. Infrared spectroscopic characterization**


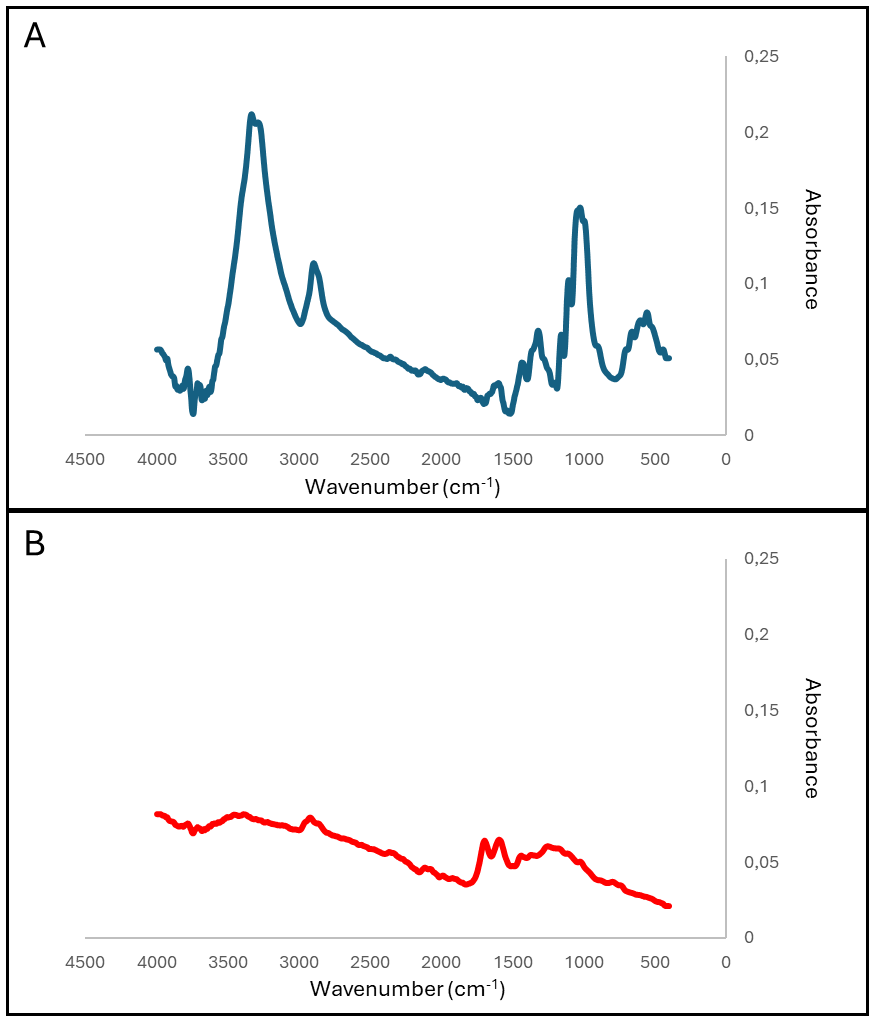


**Figure S2**. Infrared spectra of (A) raw cotton and (B) py-CFs.

**4. Study of the variables affecting the extraction process**

*4.1 Effect of the washing volume*

| 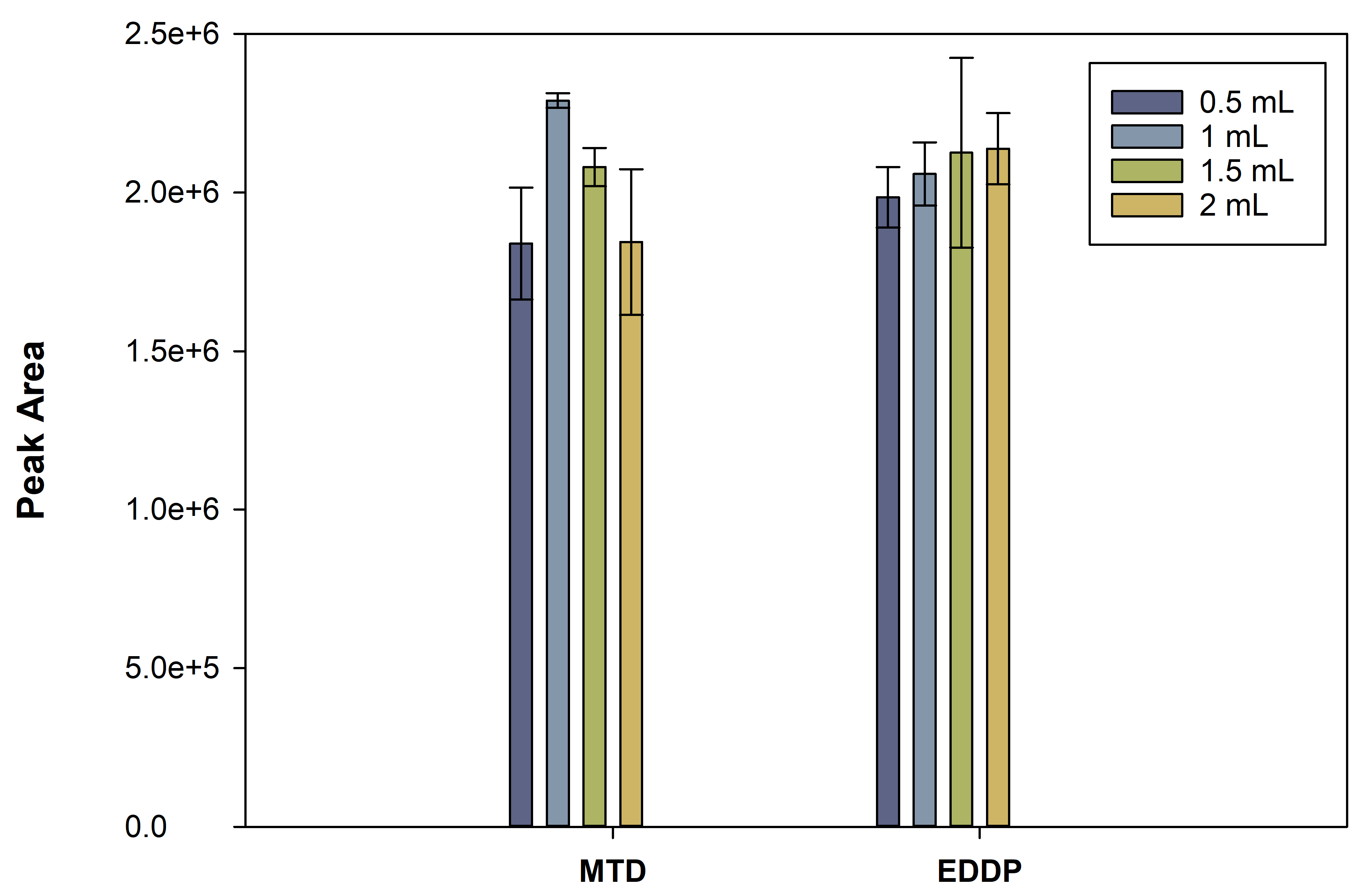 |
| --- |

**Figure S3.** Effect of the washing solution volume.

*4.2 Correlation of centrifugation speed and sample flow*

| 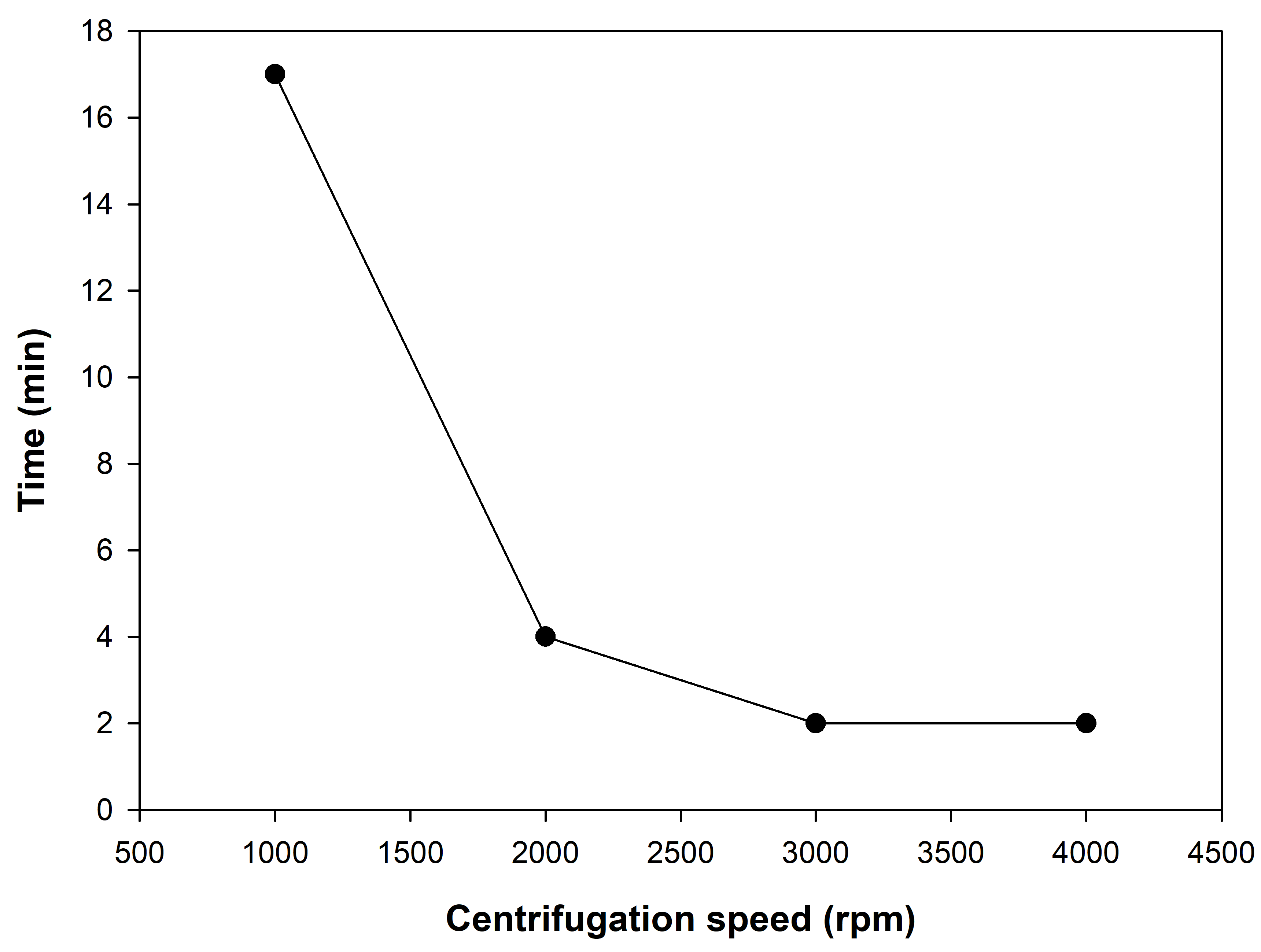 |
| --- |

**Figure S4.** Correlation between the centrifugation speed and the time necessary to completely flow the sample through the sorbent.

*4.3 Elution profile*

Two different criteria have been applied to select the most suitable solvent: i) the elution speed to reduce the time of analysis, and ii) the stability of the analytical signal. The elution speed ensures shorter time of analysis, thus affecting positively on the frequency of analysis. The selected criterion has been the time necessary to complete the elution of the analytes defined as the time to reach the baseline from the appearance of the analytical signal. The eluents have been classified from shorter to longer elution speed: isopropanol/acetonitrile (1 min), methanol/water/formic acid (1.7 min), and methanol containing formic acid (2.5 min), while methanol provides an incomplete elution behavior. On the other hand, the stability of the analytical signal relies on the reproducibility of the formation of the spray between samples. The isopropanol/acetonitrile eluent provides a smoother transient signal, while the rest of solvents present a sawtooth shape. Consequently, isopropanol/acetonitrile has been selected as the elution solvent as it presents the highest sensitivity, the shortest time of analysis, and the most stable formation of the electrospray.

| 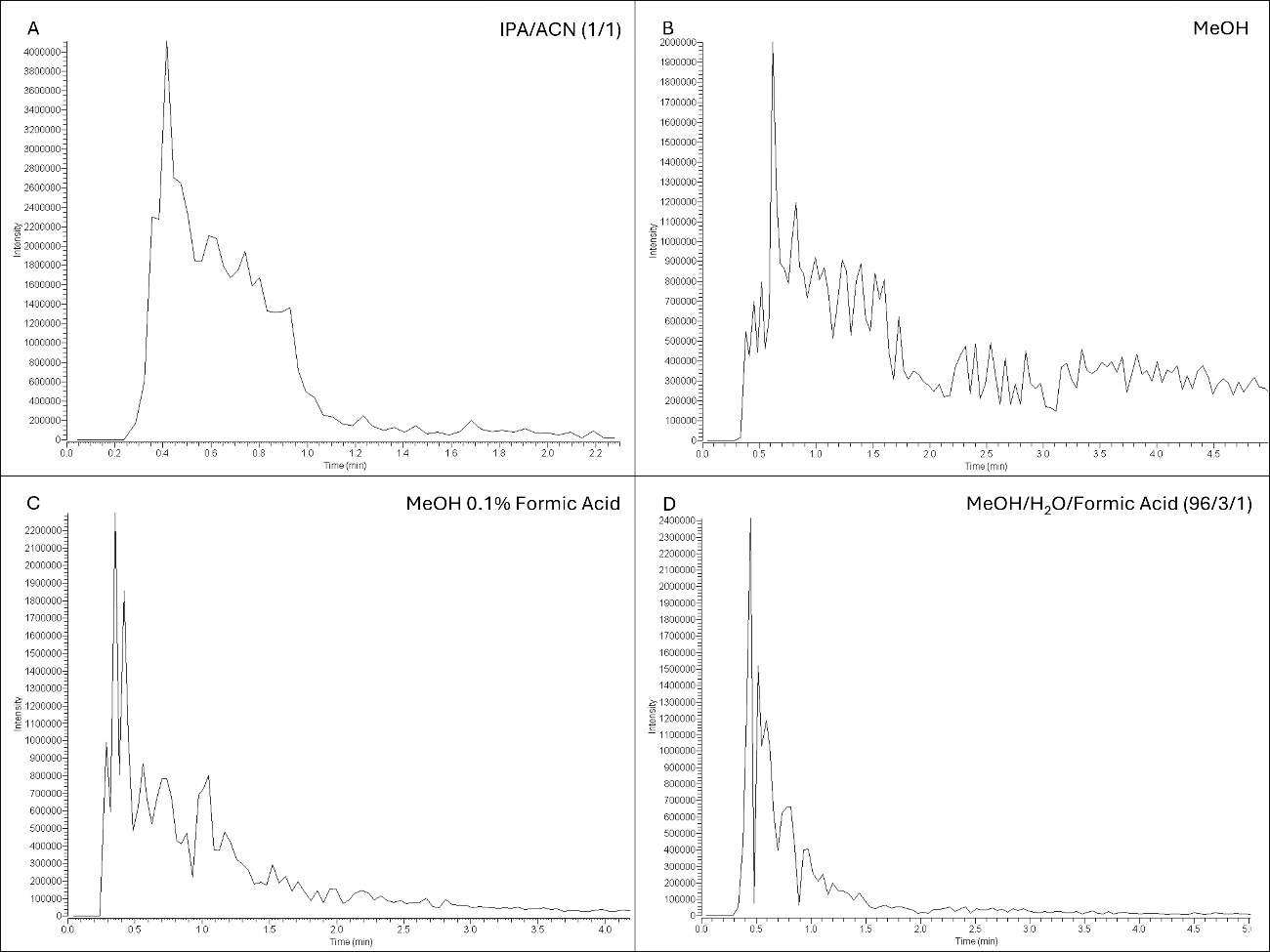 |
| --- |

**Figure S5**. Effect of the elution and ionization efficiency ability of the studied organic solvents.

**5. Study of the stability of the analytes**

| 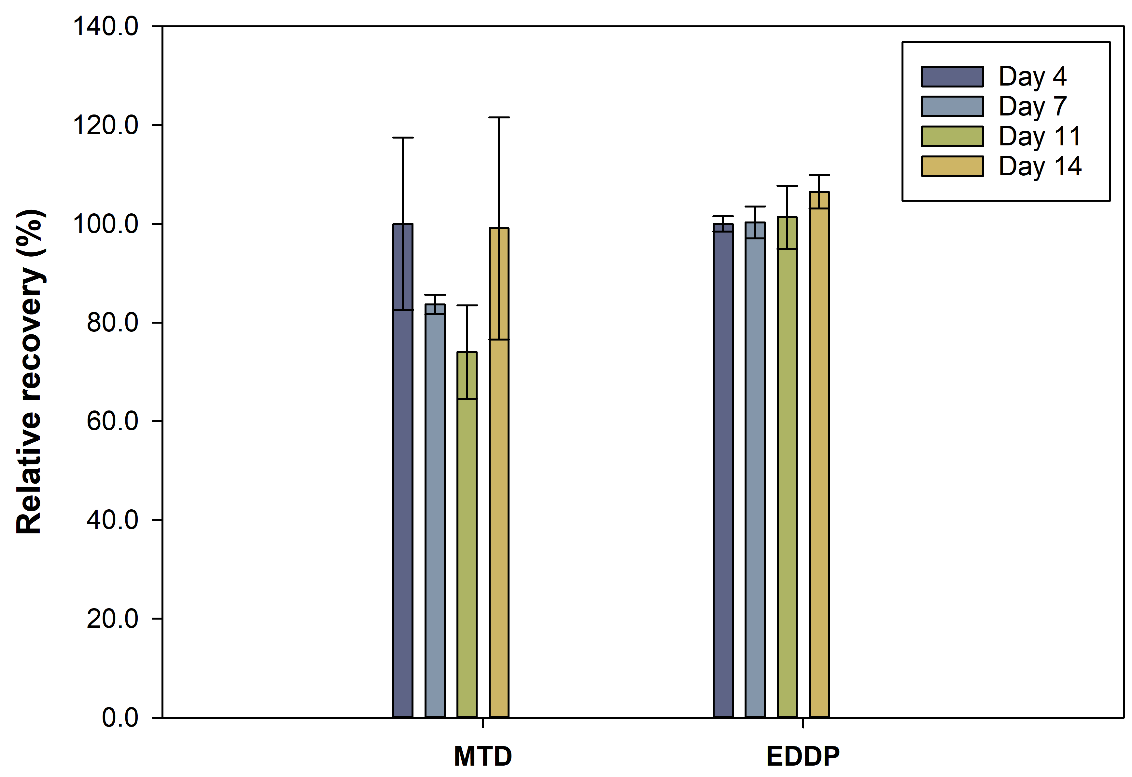 |
| --- |

**Figure S6**. Stability of the analytes after the extraction procedure prior to PT-ESI-MS/MS analysis.

**6. Chronogram of a positive urine sample**


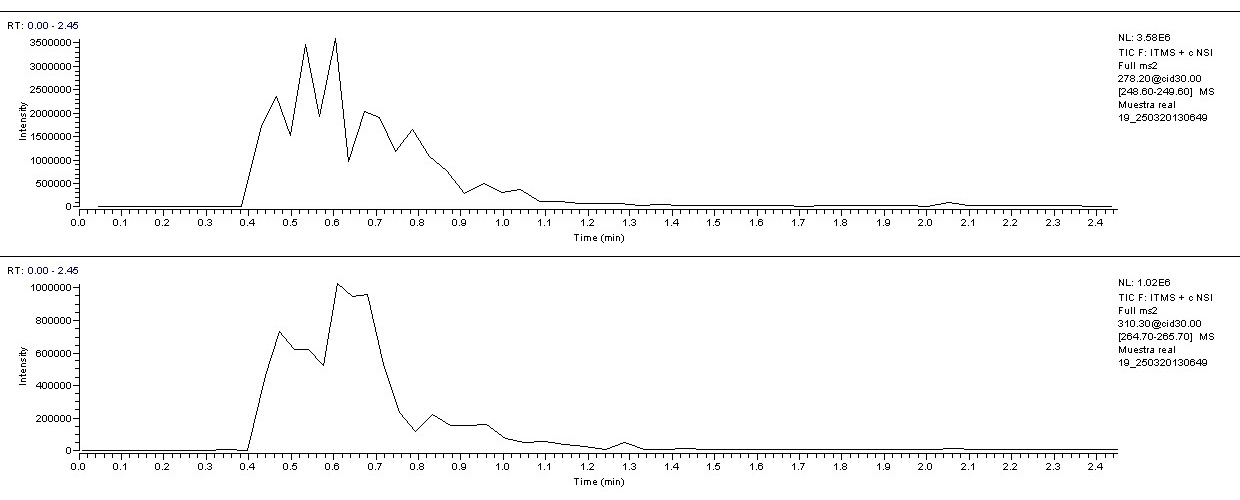


**Figure S7**. Chronogram obtained for the analysis of a positive urine sample. The quantification transitions for EDDP (upper panel) and methadone (lower panel) are shown.

**7. Evaluation of the greenness and practicality of the proposed method**

The greeness and practicality of the performance proposed analytical method were evaluated using *Sample preparation metric of sustainability* (SPMS) [1], *Analytical greeness metric for sample preparation* (AGREEPrep) [2], and *Blue applicability grade index* (BAGI) [3], respectively. The pictograms with the overall results are shown in Figure S8.

| A)  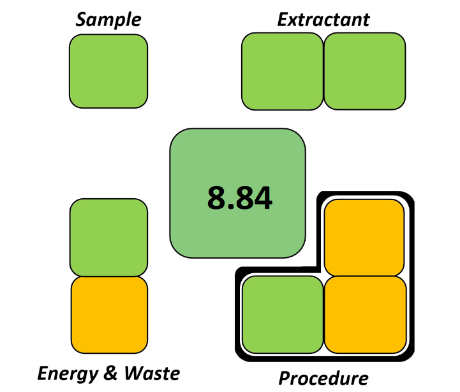 | B) 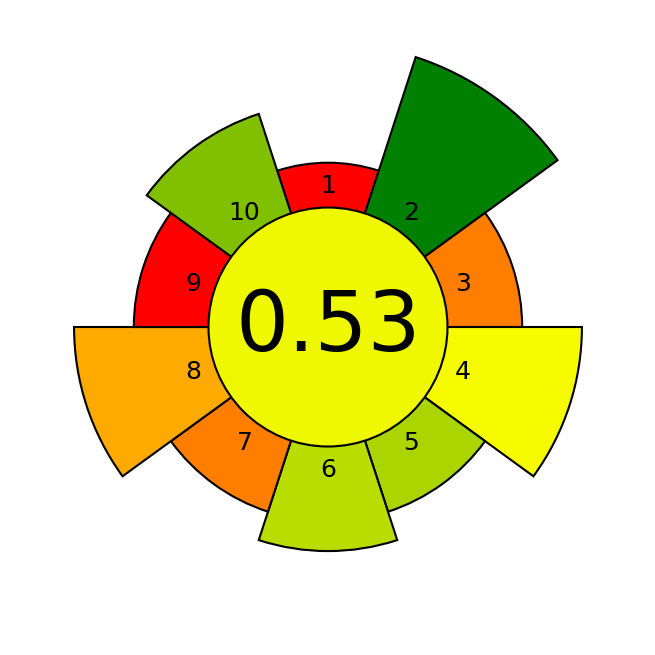 | C)  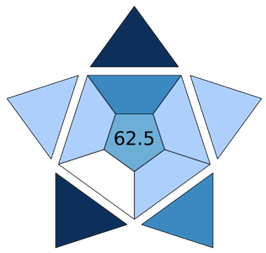 |
| --- | --- | --- |

**Figure S8**. Evaluation of the greenness and practicality of the proposed analytical method using: A) SPMS, B) AGREEPrep, and C) BAGI tools.

According to the SPMS tool (Figure S8A), the overall result (8.84 out of 10) demonstrates the grenness character of the analytical methodology, mostly based on the low sample, extractant, and waste amount, the natural origin of the extractant, and the possibility of extracting different samples simultanoeusly. However, the debilities lie on the number of steps and the time necessary to complete the extraction procedure, as well as the necessity to stir the samples by centrifugation. The AGREEPrep tool (Figure S8B)provides an overall result of 0.53 out of 1. The main strengths are the lack of hazardous materials involved in the analytical process, the reduced sample volume, the high sample throughput, and the operator safety. On the other hand, the main limitations are the *ex situ* sample preparation although an integrated elution-ionization is carried out and the advanced instrumentation that is required. In the case of BAGI (Figure S8C), the overall result (62.5 out of 100) is acceptable. In this case, the main advantages are the no need to preconcentrate the eluates and the high sample throughput. However, the limitations are related to the limited number of analytes, the semi-automation of the process, and the necessity of instrumentation that is not available in all laboratories.

**References**

1. González-Martín R, Gutiérrez-Serpa A, Pino V, Sajid M (2023) A tool to assess analytical sample preparation procedures: Sample preparation metric of sustainability. J Chromatogr A 1707:464291. https://doi.org/10.1016/j.chroma.2023.464291

2. Wojnowski W, Tobiszewski M, Pena-Pereira F, Psillakis E (2022) AGREEprep – Analytical greenness metric for sample preparation. TrAC Trends Anal Chem 149:116553. https://doi.org/10.1016/j.trac.2022.116553

3. Manousi N, Wojnowski W, Płotka-Wasylka J, Samanidou V (2023) Blue applicability grade index (BAGI) and software: a new tool for the evaluation of method practicality. Green Chem 25:7598–7604. https://doi.org/10.1039/D3GC02347H
